# EXPLORING NEW WAYS TO IMAGINE OUR FUTURE THROUGH INTERGENERATIONAL CASE STUDY PROJECTS IN ELEMENTARY SCHOOL

**DOI:** 10.1093/geroni/igad104.1129

**Published:** 2023-12-21

**Authors:** Elizabeth Bergman

**Affiliations:** Ithaca College, Ithaca, New York, United States

## Abstract

Aging education is largely absent from the PK-12 curriculum. When students do encounter messages about aging and older adults it is generally through such activities as dressing “like a 100-year-old” to celebrate the 100th day of school which promotes the internalization of ageist stereotypes starting at a young age. Motivated by a desire to push back against these ageist messages in school, the presenter partnered with elementary school educators and school administrators to develop and deliver two intergenerational case study projects involving elementary school-aged children, undergraduate students, and older adults. The presenter will highlight objectives, activities, and outcomes of the Let’s Dream Together and Ages & Stages in Our Community projects and provide suggestions for aging educators interested in similar initiatives.

